# Combination therapy with DHA and BMSCs suppressed podocyte injury and attenuated renal fibrosis by modulating the TGF-*β*1/Smad pathway in MN mice

**DOI:** 10.1080/0886022X.2022.2120821

**Published:** 2023-01-17

**Authors:** Yongzhang Li, Suzhi Chen, Jinchuan Tan, Yan Zhou, Meifang Ren, Qian Zhang, Meijiao Zhao, Guodong Yuan, Wenxi Zhang, Fengwen Yang

**Affiliations:** a Department of Urology, Hebei Province of Chinese Medicine, Shijiazhuang City, Hebei Province, China; b Department of Nephrology, Hebei Province of Chinese Medicine, Shijiazhuang City, Hebei Province, China; c Department of Pharmacy, Hebei Province of Chinese Medicine, Shijiazhuang City, Hebei Province, China

**Keywords:** Membranous nephropathy, dihydroartemisinin, bone marrow mesenchymal stem cells, podocyte injury, renal fibrosis, TGF-*β*1/Smad

## Abstract

Artemisinin has immunomodulatory, anti-inflammatory, and antifibrotic effects. Some studies have demonstrated that artemisinins have a protective effect on the kidney. DHA is a derivative of artemisinin and has effects similar to those of artemisinin. Human bone marrow-derived mesenchymal stem cells (BMSCs) accelerate renal repair following acute injury. In the study, we investigated the effects of combination therapy with DHA and BMSCs on membranous nephropathy (MN) mice. The 24-h urinary protein, serum total cholesterol (TC) and triglyceride (TG) levels, and renal histopathology, were measured to evaluate kidney damage. Anti-PLA2R, IgG, and complement 3 (C3) were detected by ELISA. The expression levels of the podocyte injury-related proteins were analyzed by immunohistochemistry. The protein expression levels of *α*-SMA, ED-1, TGF-*β*1, p-Smad2, and p-Smad3 were detected by western blot to analyze renal fibrosis and its regulatory mechanism. Results showed that combination therapy with DHA and BMSCs significantly ameliorated kidney damage in MN model mice by decreasing the levels of 24 h urinary protein, TC and TG. This combination therapy also improved renal histology and reduced the expression of IgG and C3 in the glomerulus. In addition, this combination therapy decreased the expression of podocin and nephrin and relieved renal fibrosis by downregulating *α*-SMA and ED-1. Furthermore, this combination therapy suppressed TGF-*β*1 expression and Smad2/3 phosphorylation. This result (i.e., this combination therapy inhibited the TGF-*β*1/Smad pathway) was also supported *in vitro*. Taken together, combination therapy with DHA and BMSCs ameliorated podocyte injury and renal fibrosis in MN mice by downregulating the TGF*β*1/Smad pathway.

## 1. Introduction

Membranous nephropathy (MN) is the most common cause of adult-onset nephrotic syndrome. Up to 40% of MN patients progress to end-stage renal failure, a destructive disorder that requires dialysis or kidney transplantation [1]. The diagnosis of MN depends on the characteristic pathological changes, which include the presence of immunocomplex deposits between the glomerular basement membrane (GBM) and the podocyte [2], diffuse thickening of GBM, and granular deposits of IgG and complement along the periphery of glomerular capillary loops [3]. Glomerular podocytes are highly specialized cells with a unique and complex structure that, together with the GBM and the endothelium, constitute the blood-urine filtration barrier in the glomeruli. With their large foot processes, podocytes integrate with neighboring cells and surround the glomerular capillaries in a tight cell layer [4]. Injury to podocytes leads to proteinuria and podocyte loss [5]. PLA2R binds to the corresponding antigen (anti-PLA2R) on podocytes to form immune complex, which deposits in renal epithelial cells and activates complement (e.g., C3), resulting in renal pathological damage and proteinuria generation. Research has demonstrated that podocytes are directly or indirectly damaged in nearly all types of glomerulonephritis [6]. Renal fibrosis is the final common pathway of almost all chronic kidney diseases (CKDs) [7]. In the late stage of MN, renal fibrosis is caused by the accumulation of excess extracellular matrix (ECM), which is an important indicator of the disease progression of MN. Kidney function was evaluated by blood biochemical markers TC and TG. During the pathogenic progression of MN, many cytokines (particularly TGF-*β*1), chemokines, and complement components accumulate in the kidney and cause many types of resident kidney cells and infiltrated cells to turn into myofibroblast-like cells to secrete ECM (particularly collagen) [8]. Mature TGF-*β* then signals to fibroblasts or other tissue-specific myofibroblast precursor cells by binding the receptor TGF-*β*RII on the cell surface, which forms a dimer with TGF-*β*RI. This heterodimer then phosphorylates the C-termini of Smad2 and Smad3, effector proteins that translocate to the nucleus and, along with binding partner Smad4 and other accessory proteins, bind to Smad-binding elements in the promoters of profibrotic genes, driving processes such as myofibroblast differentiation and collagen deposition.

To date, the common therapy for MN is a combination of glucocorticoids and immunosuppressive or cytotoxic agents, accompanied by drugs for other complications, including edema, hypertension, and dyslipidaemia. However, the effectiveness of these drugs in treatment of MN is limited. Thus, there is an urgent need for novel, effective and safe therapies for MN treatment. Human MSCs have been proposed as a plausible therapeutic tool, as they are easily isolated from the bone marrow and can be cultured and expanded into adequate numbers for therapeutic administration due to their ability to extensively proliferate *in vitro* [9]. In numerous models of kidney injury, the administration of bone marrow-derived mesenchymal stem cells (BMSCs) has been reported to protect against acute injury and enhance renal regeneration [10]. Although the exact mechanisms of repair have yet to be elucidated, there is substantial evidence that BMSCs elicit renal repair through endocrine and paracrine mechanisms [11]. BMSCs can also differentiate in damaged tissues and repair corresponding tissues [12]. BMSCs effectively repaired podocyte injury and promoted podocyte regeneration through paracrine BMP-7, thus delaying the development of diabetic nephropathy [11]. Artemisinin, a sesquiterpene endoperoxide derived from *Artemisia annua* L., is an antimalarial drug [13]. Researchers have synthesized a family of derivatives of artemisinin, including artesunate (ART, polar derivative), dihydroartemisinin (DHA, active metabolite), artemether, and arteether (lipid-based derivatives). In recent years, researchers have found that the effects of artemisinin and its derivatives as antagonists of the development or progression of fibrotic phenotypes have been characterized in fibrotic models across multiple tissues, suggesting the potential utility of these compounds for the treatment of several fibrotic disease states [14]. In a rat model of bleomycin-induced pulmonary fibrosis, daily i.p. administration of dihydroartemisinin (DHA) blunted the fibrotic response, subsequently decreasing collagen content in the lung [15]. Zheng et al. reported that DHA treatment inhibits the expression of TGF-*β* and blocks the TGF-*β*/Smad signaling pathway, which then inhibits the overdeposition of ECM to alleviate fibrosis [16]. Yang et al. also reported that DHA decreases the number of inflammatory cytokines and alveolar inflammation and attenuates lung injury and fibrosis in pulmonary fibrosis [17]. However, there is a lack of sufficient research on the treatment of renal fibrosis with DHA, and the specific mechanism of DHA on the progression of MN remains unclear. In addition, podocyte is one of the important components of glomerular filtration barrier and is closely related to MN progression. Artemisinin derivative SM934 inhibited proteinuria and podocyte injury in experimental MN [2]. Artemisinin protected the podocyte of Heymann nephritis rats [18].

The damage of podocyte structure and function leads to proteinuria in the late stages of disease. In the current study, we hypothesized that combination therapy with DHA and BMSCs may reduce proteinuria production by repairing damaged podocytes, and finally play a therapeutic role on MN in mice. Our study suggested that combination therapy with DHA and BMSCs suppressed podocyte injury in MN mice and attenuated tubulointerstitial fibrosis by modulating the TGF-*β*1/Smad signaling pathway.

## 2. Materials and methods

### 2.1. Animals

Female BALB/c mice (aged 6 weeks and weighing 18–22 g) were purchased from Chengdu Dashuo Biological Co., Ltd. (Sichuan, China). The mice were placed in an isolated environment with a temperature of 22 °C–24 °C and a humidity of 40%–60% in a 12 h light-dark cycle and were provided clean drinking water and chow. In this study, all animal experiments were conducted according to the ethical standards for experimental animals (Medical Ethics Committee of Hebei Provincial Hospital of Traditional Chinese Medicine, No: 2021-KY-017-01). Mouse BMSCs were purchased from Cyagen Biosciences (China, MUBMX-01001).

### 2.2. MN model

The MN mouse model was induced by cationic bovine serum albumin (C-BSA). C-BSA was prepared according to Border’s method [19], as we described previously [20]. Except for five mice randomly selected as the control group, the remaining mice were administered C-BSA for 4 weeks to establish the MN mouse model [21]. In detail, in the first week, every model mouse was subcutaneously injected with an emulsion of 0.2 mg C-BSA and isopyknic Freund’s Incomplete Adjuvant. Afterwards, mice were injected with 13 mg C-BSA into the tail vein every other day for three weeks. The 24 h urine collections were performed on individual mice in metabolic cages, and 24 h urinary total protein (UTP) was detected by the biuret method. The 24 h UTP >60 μg was used as the standard for successful preparation of the MN model, and then the MN model was identified by blood biochemical examination (TC and TG) and pathological examination (HE staining, PASM staining, and transmission electron microscopy (TEM)).

### 2.3. Animal groupings

Mice in the MN model (24 h UTP > 60 μg) were randomly divided into four groups: model group (MN model), MN + DHA group, MN + BMSCs group, and MN + DHA + BMSCs group. Specifically, MN + DHA group (10 μM DHA and gastric irrigation), MN + BMSCs group (2 × 10^6^/200 μL BMSCs suspension and tail vein injection), and MN + DHA + BMSCs group (combination of DHA and BMSCs). The same amount of saline was administered to the mice in the model and control groups at the same time. All mice were treated with the corresponding drugs once a day for 3 weeks. Then, the mice were anesthetized with sodium pentobarbital, and blood and urine samples were collected. At the endpoint of the animal experiment, all the mice were sacrificed, and the kidney tissues were collected and stored at −80 °C.

### 2.4. Biochemical analysis

The levels of total cholesterol (TC) and triglycerides (TG) in serum were detected according to the manufacturer’s instructions (Nanjing Jiancheng Bioengineering Institute, Nanjing, China).

### 2.5. Renal pathological analysis

The kidney tissues were first fixed in 10% formalin and then embedded in paraffin. Hematoxylin-eosin (H&E), Masson staining, periodic acid-silver metramine (PASM) staining, and TEM were performed on 4-μm-thick paraffin sections to observe the morphological alterations of the kidney during MN.

#### 2.5.1. Transmission electron microscope

The kidney samples were prepared. Kidney samples were fixed in cacodylate buffer (containing 2.5% glutaraldehyde and 2.5% paraformaldehyde, 0.1 M, pH7.4) for 24 h. The samples were soaked in 1% osmium tetraoxide for 1 h at 4 °C, and then graded dehydrated with absolute ethanol. Samples were embedded in Epon 812 longitudinally, then cut into ultrathin sections were at 70 nm, and compared with uranyl acetate and lead citrate. Finally, pathological changes of podocytes were observed by TEM.

### 2.6. Enzyme-linked immunosorbent assay (ELISA)

Blood collected from the abdominal aorta was centrifuged at 3000 rpm for 10 min at 4 °C to acquire serum. The 96-well plates were coated with 4 μg/mL anti-PLA2R, rabbit IgG and rabbit C3 separately in sodium carbonate buffer (pH 9.6) and incubated overnight at 4 °C. After blocking and washing, the mouse renal tissue samples, which were diluted, were added and incubated for 2 h at room temperature. After washing, an HRP-labeled sheep polyclonal antibody against mouse (1:10,000, Abcam, Cambridge, UK) was applied and incubated for 1 h. Then, the substrate was added, and absorbance was detected at 450 and 570 nm with an ELISA reader (Thermo, USA).

### 2.7. Immunohistochemistry

As previously mentioned, paraffin sections of renal tissues were dehydrated successively in a graded alcohol series (75%, 85%, 95%, and 100%, v/v). Then, sections were washed three times with PBS and soaked in 3% H2O2 deionized water over a period of 20 min. The sections were then washed with PBS again followed by blockade with goat serum for 15 min. Next, the sections were incubated with primary antibodies against nephrin (ab216341, 1:1000, Abcam, USA) and podocin (ab50339, 1:1000; Abcam, USA) at 4 °C overnight. After the sections were incubated with secondary antibody at 37 °C for 2 h, they were stained with DAB for 5–7 min. PBS rinses were performed for 5 min, and the slices were restained with hematoxylin for 2 min, followed by treatment with fractionated alcohol in hydrochloric acid and were then rinsed in running water for 10 min. The slices were dried naturally and sealed, and finally, the positive expression of proteins was observed under an optical microscope, and the slices photographed for recording. Immunohistochemical semiquantitative analysis was performed. Five fields (×400) were randomly selected from each section. ImagePro Plus 6.0 software was used to calculate the integral absorbance and area of positive results under each field, and the integral absorbance/area was used as the semi-quantitative results of the detection index.

### 2.8. Western blot

First, the kidney tissues were homogenized. Then, proteins were extracted by using RIPA lysis buffer (Abcam, Ab156034, UK). The protein concentration was determined by a Pierce™ BCA Protein Assay Kit (Thermo Scientific). The protein extracts were separated by SDS–PAGE electrophoresis and then transferred onto PVDF (polyvinylidene fluoride) membranes. The membrane was blocked with 5% skim milk powder (diluted with Tris-buffer) for 2 h and then incubated overnight at 4 °C with primary antibodies against *α*-SMA (ab7817, 1:1000, Abcam, USA), ED-1 (qy-1088R, 1:1000, Santa Cruz, USA), TGF-*β*1 (ab215715, 1:500, Abcam, USA), p-Smad2 (ab280888, 1:1000, Abcam, USA), and p-Smad3 (ab63403, 1:1000, Abcam, USA). Subsequently, a secondary goat anti-rabbit IgG (HRP) antibody (ab205718, 1:2000, Abcam, USA) was applied and incubated with the membranes at room temperature for 1 h. The blots were assayed by using the Novex™ ECL Chemiluminescent Substrate Reagent Kit (Thermo Fisher Scientific™, WP20005, USA). *β*-actin was used as an internal reference protein. Finally, ImageJ software was used to analyze the results.

### 2.9. Cck-8 analysis

The fibrosis was induced by TGF-*β*1 (10 ng/mL) in their cell culture model. Mouse podocytes (Otwo Biotech, China) were cultured in RPMI-1640 medium containing 10% fetal bovine serum and divided into the control group, a MN group, a MN model + SB431542 group, a MN + DHA + BMSCs group, and a MN + DHA + BMSCs + SB431542 group. The cells were cultured at 37 °C in 5% CO2 for 24 h. Mouse podocytes at the logarithmic growth stage were inoculated into 96-well plates at a density of 5 × 10^3^ cells per well. The concentration of SB431542 was 5 μM. The SB431542 was pretreated for 2 h, and the total exposure time was 26 h for the cell culture experiments. The concentration of DHA was 10 μM, and the total exposure time was 24 h for the cell culture experiments. BMSCs (2 × 10^6^/mL) were seeded into conventional 6-well plates, incubated for 24 h, culture supernatant collected, filtered through a 0.22 m filter and stored in –20 °C refrigerator for reserve. The concentration of BMSCs was 2 × 10^6^/mL, and the total exposure time was 24 h for the cell culture experiments. After 24 h of culture, 10 μL of CCK8 solution was added to each well and cultured at 37 °C for 4 h. The optical density of each well was measured at 450 nm, and the cell survival rate was calculated.

### 2.10. Statistical analysis

The experimental data were analyzed with SPSS 17.0 statistical software and GraphPad Prism 7 software. All the data are expressed as the mean ± standard error (mean ± SD). One-way ANOVA (one-way analysis of variance) was used to compare more than two groups, the LSD-*t* test (least significant digital) was used for homogeneity of variance, and Dunnett’s *t* test was used for heterogeneity of variance. The *p* values < .05 were considered to be significant.

## 3. Results

### 3.1. Protective effect of the combination therapy with DHA and BMSCs on the kidneys of MN mice

**Figure 1.**
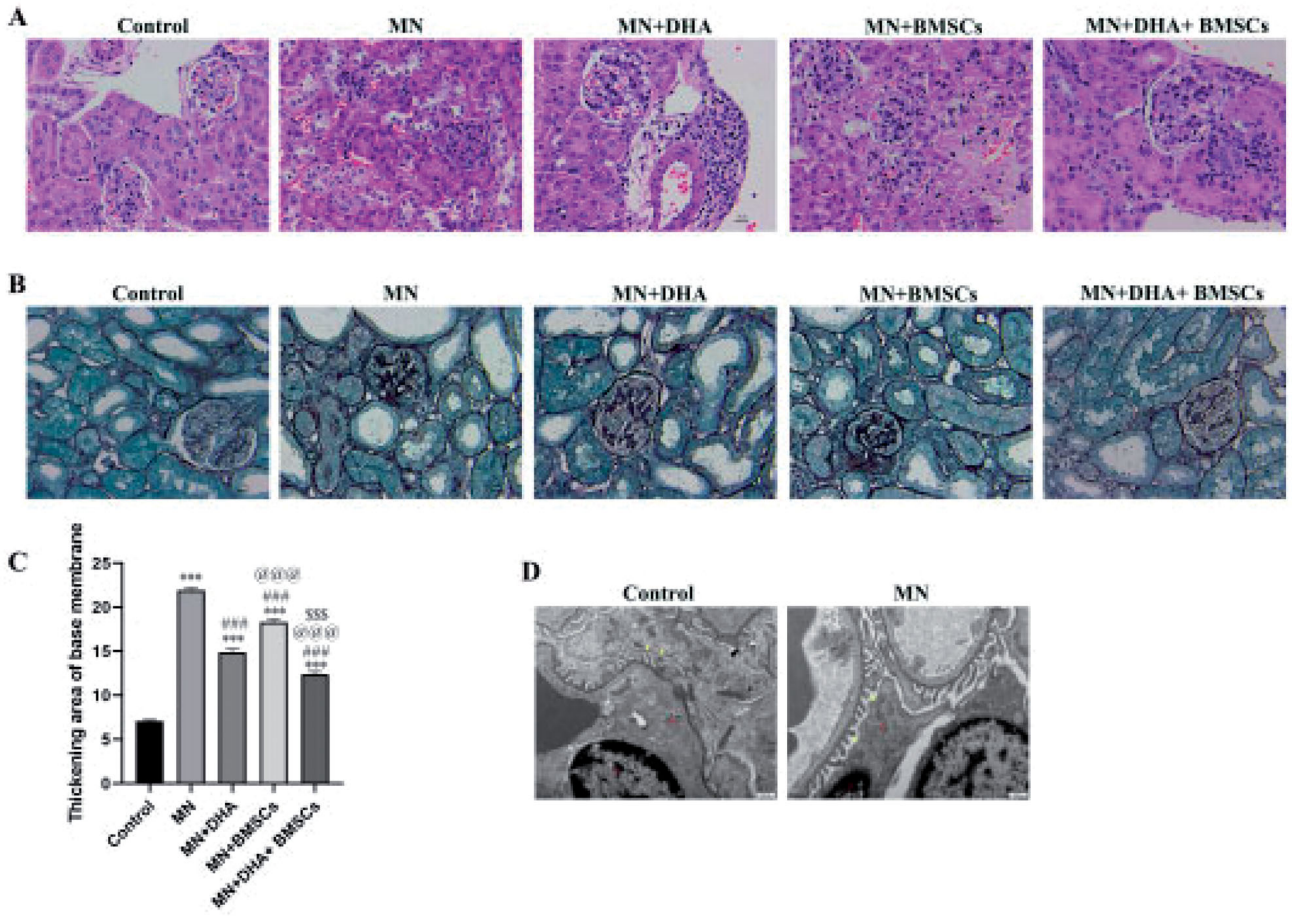
Identification of the MN mouse model. (A) H&E staining showing histopathological changes in renal tissue (original magnification ×400; scale bar 10 μm). (B,C) PASM staining showing a thickened area of the basal membrane of the glomerulus and renal tubule. (D) TEM ultrastructure of renal tissue (original magnification ×6000, scale bar 500 nm). Red: mitochondria (Mi), nucleus (N). Yellow: foot process. Values are expressed as the mean ± SD, *n* = 5 per group. *p < .05 versus control group; ***p < .001 versus control group; ^#^*p* < .05 versus MN model group; ^###^*p* < .001 versus MN model group; ^@^*p* < .05 versus MN + DHA group; ^@@@^*p* < .001 versus MN + DHA group; ^$^*p* < .05 versus MN + BMSCs group; ^$$$^*p* < .001 versus MN + BMSCs group.

To evaluate the effects of DHA and BMSCs on C-BSA-induced MN model mice, 24 h UTP, TC, TG, and anti-PLA2R were detected, and renal pathological changes were observed. The results showed that the levels of 24 h UTP, TC, TG, and anti-PLA2R in the MN group were significantly higher than those in the control group, and body mass and kidney mass were significantly decreased (Table 1). HE staining suggested that the glomerular structure was intact and the cyst size was normal in the control group, while glomerular volume was increased in the MN group (Figure 1(A)). PASM staining indicated no abnormal basement membrane in the control group, while the basement membrane was thickened to different degrees and a ‘spike’ was formed in the MN group (Figure 1(B)). TEM results revealed that the podocellular structure was clear and no obvious abnormality was observed in the control group, while a large amount of electron-dense substance was deposited, and the epithelial cell foot protruded and fused extensively or disappeared in the MN group (Figure 1(C)). These results proved that the MN model was successfully established. After treatment with DHA and BMSCs, the levels of UTP, TC, TG, and anti-PLA2R decreased, and the pathological injury of renal tissue was improved. Moreover, the effect of combination therapy with DHA and BMSCs was stronger than that of single treatment (Table 1 and Figure 1(A–C)).

**Table 1.** Levels of UTP, TC, TG, Anti-PLA2R, body mass, and kidney mass.

Groups	UTP (μg)	TC (mmol/L)	TG (mmol/L)	Anti-PLA2R (ng/mL)	Body mass (g)	Kidney mass (g)
Control	13.06 ± 3.34	0.98 ± 0.26	0.71 ± 0.14	0.22 ± 0.05	39.52 ± 1.85	1.74 ± 0.11
MN	105.6 ± 15.43*	3.54 ± 0.39*	3.30 ± 0.17*	0.60 ± 0.06*	23.66 ± 1.27*	1.04 ± 0.08*
MN + DHA	74.80 ± 10.41*^#^	2.08 ± 0.22*^#^	2.04 ± 0.24*^#^	0.43 ± 0.05*^#^	26.47 ± 1.33*^#^	1.16 ± 0.01*^#^
MN + BMSCs	67.78 ± 7.53*^#^	2.55 ± 0.07*^#^	2.89 ± 0.19*^#^	0.51 ± 0.02*^#^	27.52 ± 2.31*^#^	1.21 ± 0.04*^#^
MN + DHA + BMSCs	50.11 ± 2.26*^#@$^	1.48 ± 0.17*^#@$^	1.26 ± 0.23*^#@$^	0.37 ± 0.04*^#@$^	33.26 ± 2.53*^#@$^	1.46 ± 0.12*^#@$^

*p < .05 versus control group; ^#^*p* < .05 versus MN model group; ^@^*p* < .05 versus MN + DHA group; ^$^*p* < .05 versus MN + BMSCs group.

### 3.2. Effect of combination therapy with DHA and BMSCs on immune injury in MN mice

**Figure 2.**
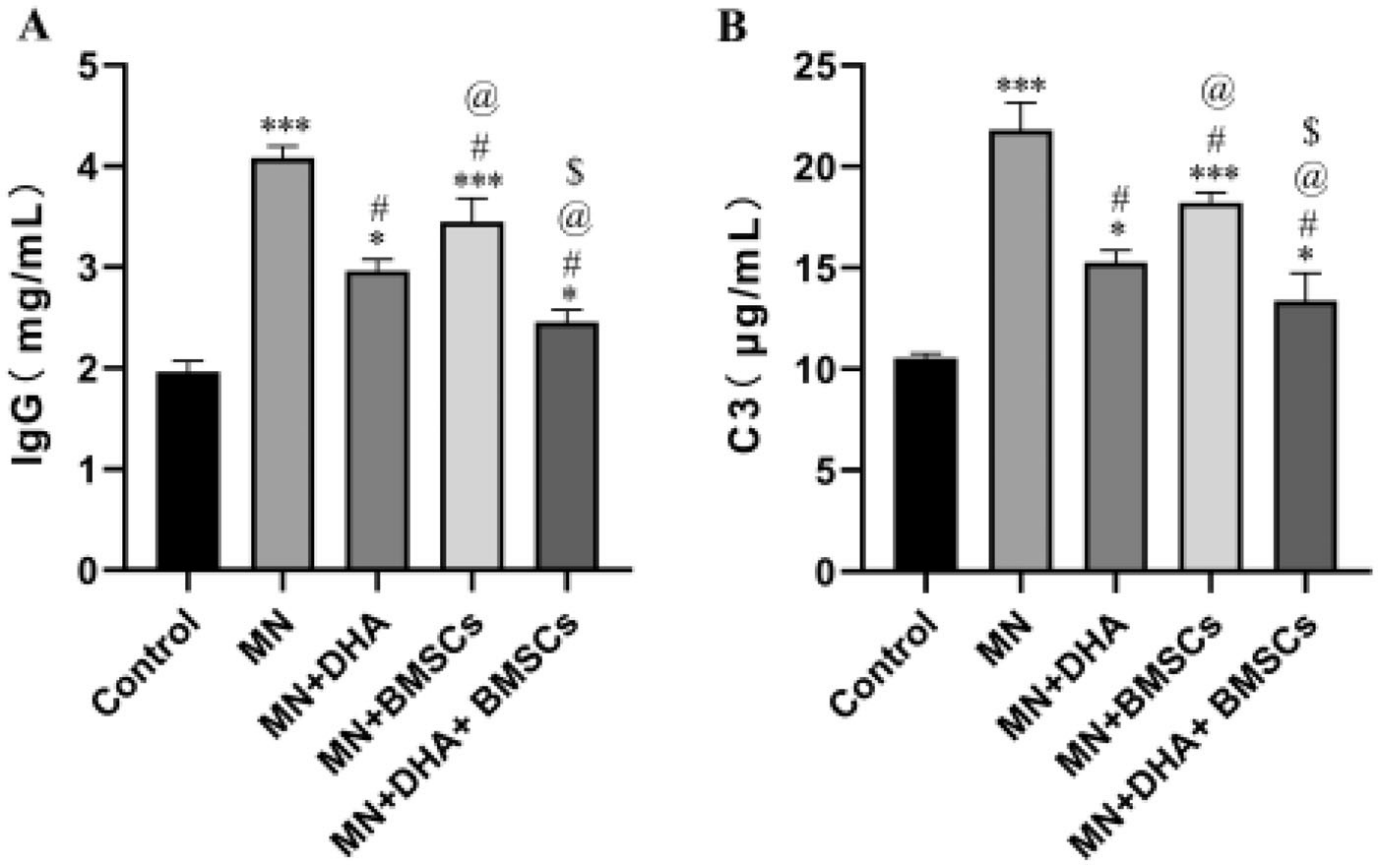
Effect of combination therapy with DHA and BMSCs on immune injury in MN mice. (A) ELISA for IgG deposition. (B) ELISA for C3 deposition. Values are expressed as the mean ± SD, *n* = 5 per group. *p < .05 versus control group; ***p < .001 versus control group; ^#^*p* < .05 versus MN model group; ^@^*p* < .05 versus MN + DHA group; ^$^*p* < .05 versus MN + BMSCs group.

The complement system plays an important role in disease progression, such as deteriorating the glomerular filtration barrier and inducing renal fibrosis. In MN, C3 and C5b-9 depositions in the kidney are typical, and they are also found in passive Heymann nephritis (PHN) animal kidneys [22,23]. Because rabbit antiserum was injected into mice, the immune system of mice could recognize these heterologous antigens and produce high levels of mouse anti-rabbit antibodies. In our experiment, IgG and C3 deposition in the glomerulus was detected by ELISA. In the model group, IgG and C3 were particularly expressed along the capillary walls in glomeruli, which is characteristic of MN. Mice in the DHA and BMSCs groups showed lower expression of IgG and C3 than the model group, suggesting that DHA and BMSCs could alleviate immune injury in MN mice. Furthermore, treatment with the combination with DHA and BMSCs lessened IgG and C3 deposition to a greater extent than either therapy alone (Figure 2(A,B)).

### 3.3. Effect of combination therapy with DHA and BMSCs on podocyte injury in MN mice

**Figure 3.**
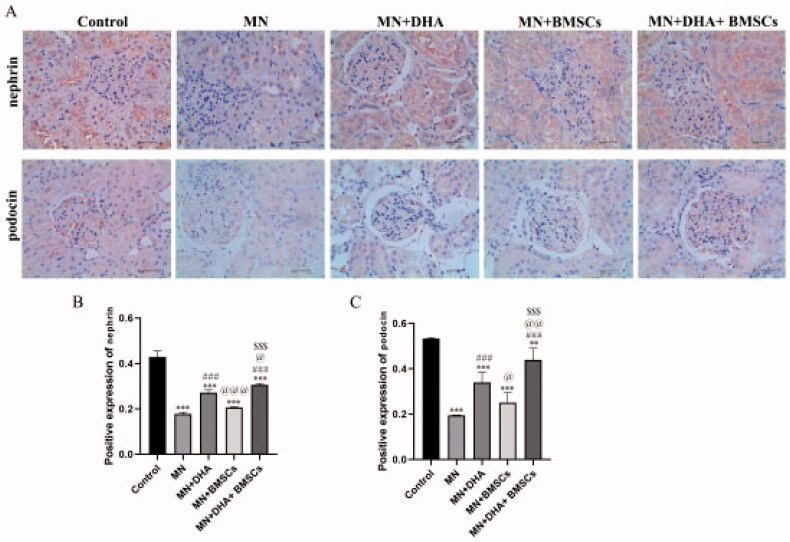
Effect of combination therapy with DHA and BMSCs on podocyte injury in MN mice. (A) Immunohistochemistry for nephrin and podocin (scale bar = 40 μm). (B) Positive nephrin expression. (C) Positive expression of podocin. Values are expressed as the mean ± SD, *n* = 5 per group. **p < .01 versus control group; ***p < .001 versus control group; ^###^*p* < .001 versus MN model group; ^@^*p* < .05 versus MN + DHA group; ^@@^*p* < .01 versus MN + DHA group; ^@@@^*p* < .001 versus MN + DHA group; ^$$$^*p* < .001 versus MN + BMSCs group.

Podocin and nephrin are important proteins expressed on the slit diaphragm and the cell bodies of podocytes, respectively, and they play central roles in maintaining the normal morphology and function of podocytes [24]. In our study, the expression levels of nephrin and podocin were reversed in the model group compared with the control group. After intervention with combination therapy with DHA and BMSCs compared with either therapy alone, the expression levels of nephrin and podocin were significantly increased (Figure 3(A–C)). These data demonstrated that combination therapy with DHA and BMSCs protects against podocyte injury in MN mice.

### 3.4. Effects of combination therapy with DHA and BMSCs on renal fibrosis in MN mice

**Figure 4.**
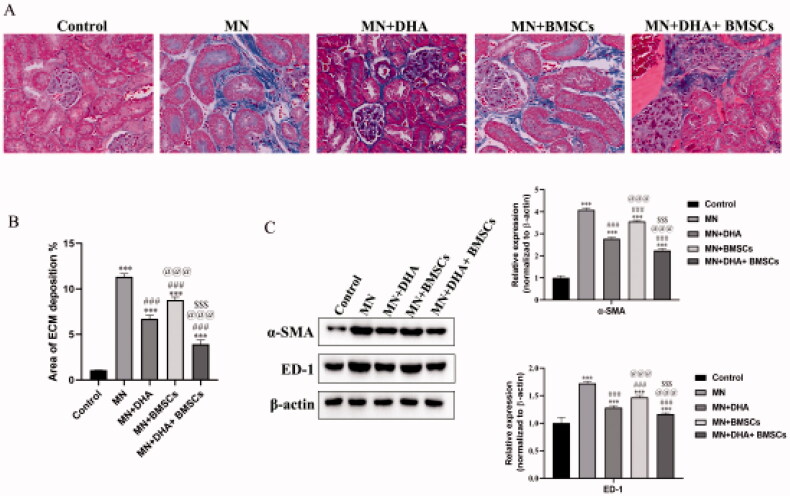
Effects of combination therapy with DHA and BMSCs on renal fibrosis in MN mice. (A) Representative sections of kidney stained with Masson’s trichrome (original magnification ×400). (B) Area of ECM deposition in the kidney. (C) Western blot of *α*-SMA and ED-1 expression in the kidney. Values are expressed as the mean ± SD, *n* = 5 per group. ***p < .001 versus control group; ^###^*p* < .001 versus MN model group; ^@@@^*p* < .001 versus MN + DHA group; ^$$$^*p* < .001 versus MN + BMSCs group.

Renal fibrosis is the primary manifestation at the end stage of MN. ECM deposition is the hallmark of renal fibrosis. Masson’s trichrome staining showed that MN mouse kidneys were filled with masses of ECM compared to the control group, and the animals receiving the combination therapy with DHA and BMSCs treatment were in much better condition than animals receiving either therapy alone (Figure 4(A,B)). In addition, the expression levels of the interstitial myofibroblast marker *α*-SMA and ED-1-positive monocytes/macrophages were examined. Western blot results showed that the protein expression levels of *α*-SMA and ED-1 were markedly increased in the MN group. As expected, combination therapy with DHA and BMSCs significantly reduced the expression of the above proteins compared with either therapy alone (Figure 4(C)). Together, these results demonstrate that in the MN model, the combination with DHA and BMSCs attenuated established renal fibrosis to a greater extent than either therapy alone.

### 3.5. Effects of combination therapy with DHA and BMSCs on the TGF-β1/Smad signaling pathway in MN mice

**Figure 5.**
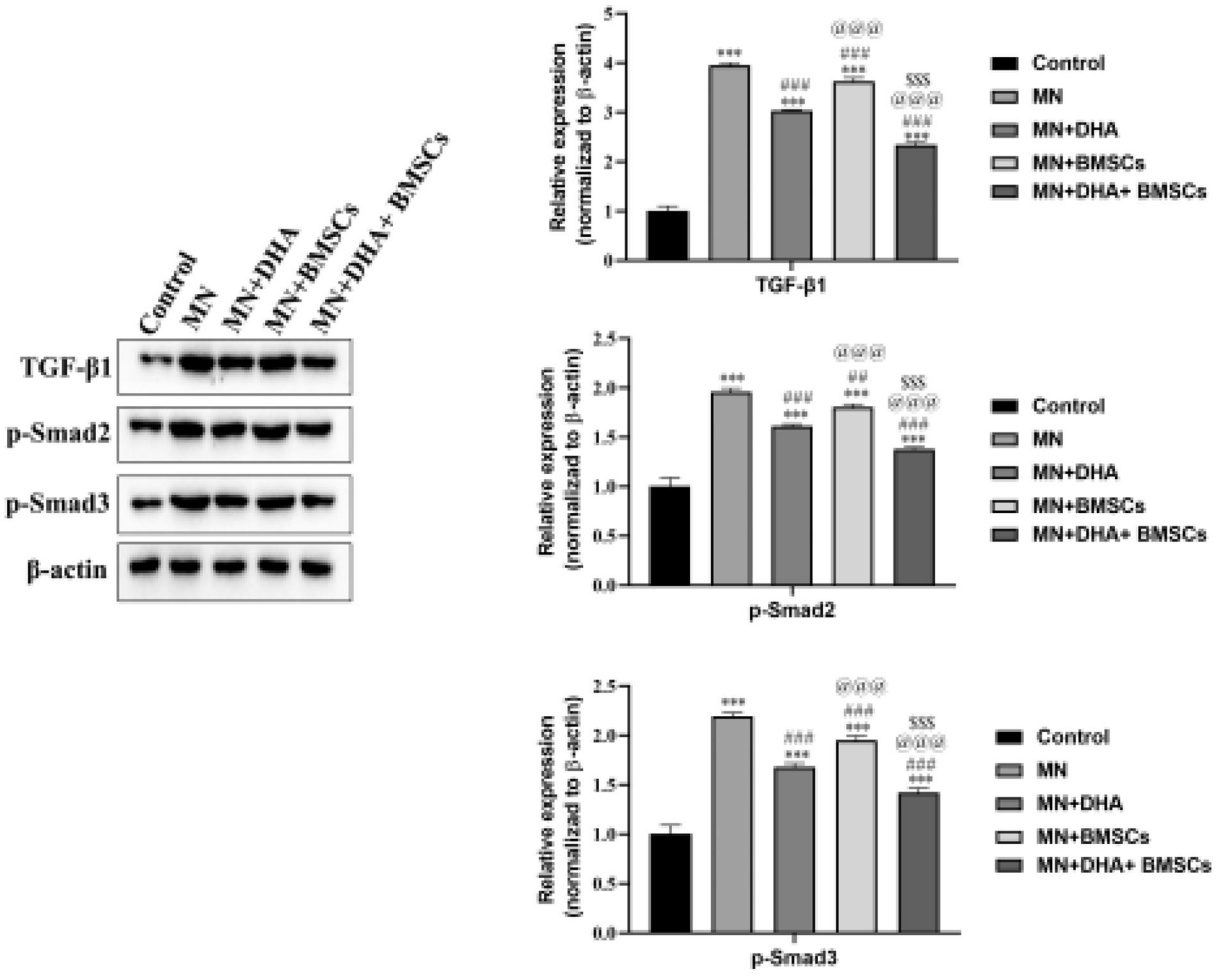
Effects of combination therapy with DHA and BMSCs on the TGF-*β*1/Smad signaling pathway in MN mice. Western blotting was applied to detect the protein expression of TGF-*β*1, p-Smad2 and p-Smad3 in the kidney. Values are expressed as the mean ± SD, *n* = 5 per group. ***p < .001 versus control group; ^##^*p* < .01 versus MN model group; ^###^*p* < .001 versus MN model group; ^@@@^*p* < .001 versus MN + DHA group; ^$$$^*p* < .001 versus MN + BMSCs group.

The TGF-*β*1/Smad signaling pathway is one of the most important profibrotic pathways in renal fibrosis. Smad2 and Smad3 proteins have been identified to be phosphorylated by activated TGF-*β* receptors, and the inhibitory Smad protein Smad7 is degraded under disease conditions [7]. To reveal the potential mechanism of the antifibrotic property of the combination therapy with DHA and BMSCs, we examined the expression of TGF-*β*1 and its downstream Smad signaling pathway. Western blot analysis showed that the protein expression levels of TGF-*β*1, p-Smad2, and p-Smad3 were markedly increased in the MN group compared with the control group. After intervention with combination therapy with DHA and BMSCs compared with either therapy alone, the expression levels of TGF-*β*1, p-Smad2, and p-Smad3 were significantly reduced (Figure 5). Taken together, the results suggested that combination therapy with DHA and BMSCs inhibited the TGF-*β*1/Smad pathway.

### 3.6. Effects of combination therapy with DHA and BMSCs on the TGF-β1/Smad signaling pathway in mouse podocytes *in vitro*

**Figure 6.**
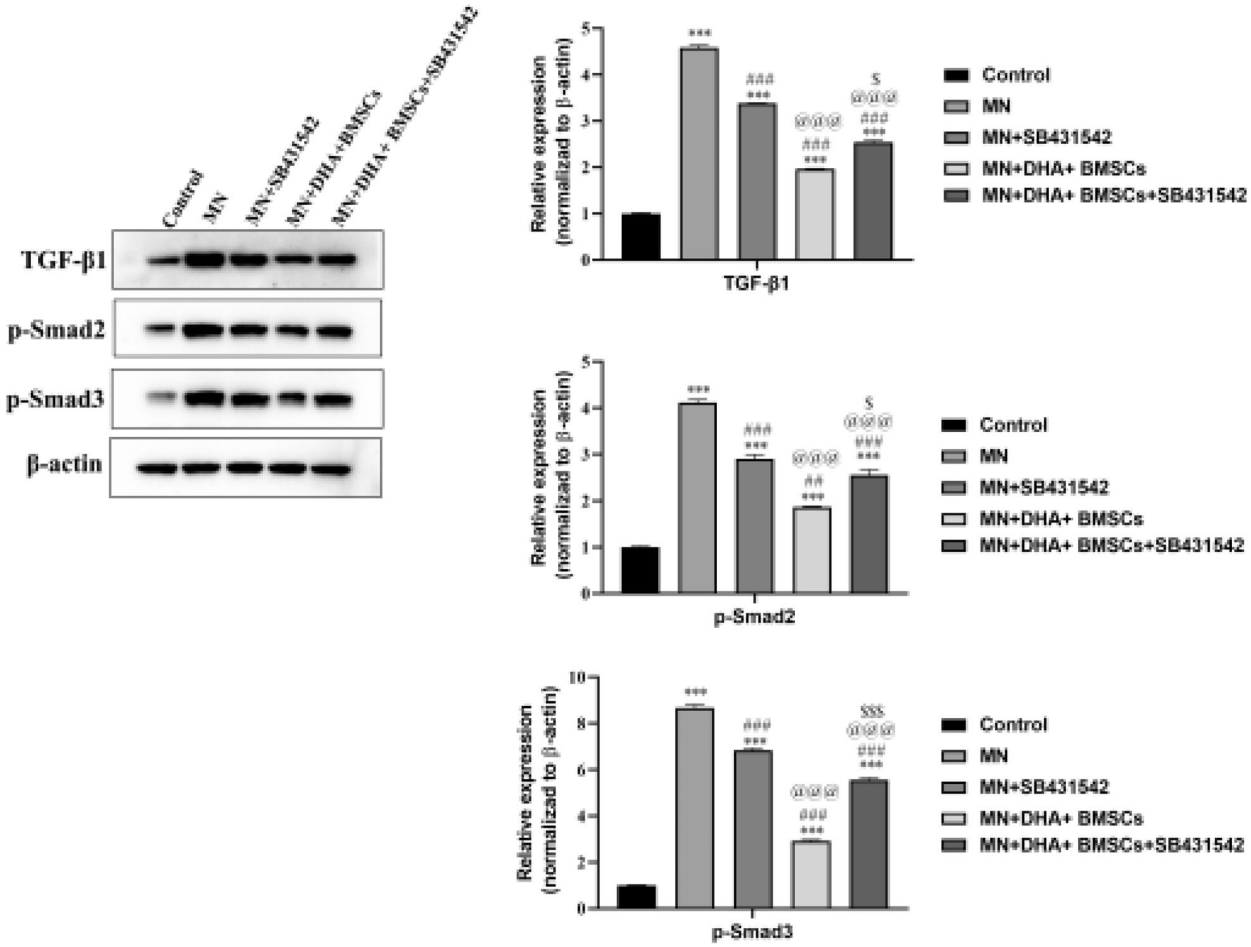
Effects of combination therapy with DHA and BMSCs on the TGF-*β*1/Smad signaling pathway in mouse podocytes *in vitro*. Western blotting was applied to detect the protein expression of TGF-*β*1, p-Smad2 and p-Smad3 in mouse podocytes *in vitro*. Values are expressed as the mean ± SD, *n* = 5 per group. ***p < .001 versus control group; ^###^*p* < .001 versus MN model group; ^@@@^*p* < .001 versus MN + SB431542 group; ^$^*p* < .05 versus MN + DHA + BMSCs group; ^$$$^*p* < .001 versus MN + DHA + BMSCs group.

SB431542 is a TGF-*β*R inhibitor that blocks the phosphorylation process of Smad2/3 by inhibiting the activation of ALK4, ALK5, and ALK7 [25]. It is found that SB431542 can prevent and delay fibrosis in lung, kidney, eye, and other organs. To explore the mechanism of the combination therapy with DHA and BMSCs in inhibiting renal fibrosis, mouse podocyte fibrosis was induced *in vitro*, and SB431542 intervention was administered to prove that the combination therapy with DHA and BMSCs inhibited renal fibrosis through the inhibitory TGF-*β*1/Smad pathway. CCK-8 analysis showed that the survival rate of mouse podocytes decreased significantly in the MN group. In the case of SB431542 intervention, the survival rate of mouse podocytes was significantly increased by combination therapy with DHA and BMSCs (Table 2). Western blot analysis suggested that the protein expression levels of TGF-*β*1, p-Smad2, and p-Smad3 were markedly increased in the MN group compared with the control group. After SB431542 intervention, the expression levels of TGF-*β*1, p-Smad2, and p-Smad3 were significantly decreased by combination therapy with DHA and BMSCs (Figure 6). Therefore, the results showed that combination therapy with DHA and BMSCs suppressed renal fibrosis by inhibiting the TGF-*β*1/Smad pathway.

**Table 2.** Effects of combination therapy with DHA and BMSCs on mouse podocyte proliferation *in vitro.*

Groups	Survival rate (%)
Control	100.00 ± 0.00
MN	72.36 ± 8.42*
MN + SB431542	80.54 ± 9.53*^#^
MN + DHA + BMSCs	90.062 ± 7.27*^#^
MN + DHA + BMSCs + SB431542	85.16 ± 5.16*^#@$^

*p < .05 versus control group; ^#^*p* < .05 versus MN model group; ^@^*p* < .05 versus MN + SB431542 group; ^$^*p* < .05 versus MN + DHA + BMSCs group.

## 4. Discussion

MN is a common glomerular disease with a significant increase in incidence. Lowering blood pressure and reducing edema are at the core of MN management, as the use of hormones and immunosuppression may cause unnecessary harm to patients. Currently, there are no specific drugs available for the treatment of MN. Therefore, it is imperative to seek safer and more effective therapeutic drugs and methods. BMSCs are a class of adult stem cells with multidirectional differentiation potential and can be used to repair kidney tissue damage. Artemisinin and its derivatives (DHA) have potential antifibrotic value and may present a new therapeutic approach for fibrosis. In this study, an MN mouse model induced by C-BSA was used to evaluate the effects of combination therapy with DHA and BMSCs on podocyte injury and renal fibrosis. The results showed that combination therapy with DHA and BMSCs markedly ameliorated immune injury, podocyte injury, and renal fibrosis and regulated the TGF-*β*1/Smad pathway.

Rat PHN is the most commonly used rodent model of human MN. PLA2R (phospholipase A2 receptor) and THSD7A (thrombospondin Type-1 domain-containing 7A) are two major pathogenic target antigens that lead to MN. The pathogenic mechanism and clinical manifestations of rat PHN are similar to MN in humans [23]. PHN is induced by passive administration of anti-Fx1A serum/antibody, which can bind to antigens on the podocyte. The deposition of immune complexes causes glomerular filtration barrier deterioration, inflammatory cell infiltration, and ECM accumulation, leading to heavy proteinuria and renal fibrosis [26,27]. These immunocomplex deposits include immunoglobulin, complement components (C3 and C5b-9), and recently identified autologous antigens [28,29]. In this study, after treatment with a combination therapy with DHA and BMSCs, UTP, TC, TG, anti-PLA2R, IgG and C3 were markedly inhibited, and histopathological changes in renal tissue were alleviated in MN mice.

Podocytes are terminally differentiated cells in the glomerulus with regularly spaced foot processes that control glomerular filtration *via* the slit diaphragm [30]. Persistent podocyte injury results in podocyte loss and death, leading to progressive kidney damage and ultimately kidney failure [31]. Indeed, podocyte injury enhanced the increase in proteinuria; as a result, podocytes are believed to be the primary target of MN. Podocin and nephrin are the main components of the slit diaphragm, which play key roles in maintaining filtration barrier integrity [32]. In the present study, we found that combination therapy with DHA and BMSCs decreased the expression of podocin and nephrin. These results suggested that combination therapy with DHA and BMSCs has the potential to protect podocytes during kidney injury.

Proteinuric states tend to initiate the intrarenal inflammatory response and accelerate renal fibrogenesis [33,34]. In the late stage of human MN, inflammatory cell infiltration and renal fibrosis are very common. The histopathological sections demonstrated that combination therapy with DHA and BMSCs markedly attenuated both interstitial inflammatory cell infiltration (especially ED-1-positive macrophages) and tubular damage in MN mice. Fibrogenesis is characterized by the accumulation of excessive ECM, and myofibroblasts are the principal source of ECM. Combination therapy with DHA and BMSCs reduced the expression of *α*-SMA and ED-1. Therefore, these findings demonstrated that combination therapy with DHA and BMSCs inhibited renal fibrosis in MN mice.

Furthermore, we tried to explore the anti-fibrosis mechanism of combination therapy with DHA and BMSCs. Although numerous different fibrogenic factors have been documented, including various cytokines and hormonal and metabolic factors, increasing evidence shows that TGF-*β* and its downstream Smad signaling play a central role in the pathogenesis of renal fibrosis in both human and experimental models of MN [35,36]. TGF-*β* recruits and activates transforming growth factor-*β* receptor to phosphorylate Smad2/3 [37], which then forms a complex with Smad4 and ultimately binds to the target gene and induces the transcription of profibrotic molecules [38]. Li et al. reported that SM934 treatment alleviates renal fibrosis by inhibiting the TGF-*β*/Smad signaling pathway [2]. SM934 treatment downregulated the expressions of TGF-*β* and Smad2/3 by reducing macrophage infiltration, and ultimately reduced the number of myofibroblasts and the expression of collagen. In the current study, we observed that combination therapy with DHA and BMSCs downregulated the expression of TGF-*β*1, p-Smad2, and p-Smad3. This result (i.e., combination therapy with DHA and BMSCs inhibited the TGF-*β*1/Smad pathway) was also supported *in vitro*. SB431542 is a small molecule inhibitor. SB431542 blocked the transmembrane transduction of TGF-*β*1 signaling by inhibiting the activation of ALK5 and blocked the phosphorylation of Smad2 and Smad3 by inhibiting the activation of ALK4 and ALK7. After SB431542 intervention, the expression levels of TGF-*β*1, p-Smad2, and p-Smad3 were significantly reduced by combined DHA and BMSCs treatment. In addition, macrophages are the main source of TGF-*β*1 in renal fibrosis, which has been well documented [39]. In the current study, the expression of ED-1, a marker of mouse macrophages, was significantly reduced after combination therapy with DHA and BMSCs treatment in MN mouse kidneys. According to the effect of combination therapy with DHA and BMSCs on renal fibrosis, we conclude that combination therapy with DHA and BMSCs is able to restrict TGF-*β*1 expression by reducing macrophage infiltration. Then, PLA2R is a transmembrane glycoprotein, which can combine with the corresponding antigens on podocytes to form immune complexes and deposit in renal epithelial cells, so as to activate complement [40]. After a series of reactions, it leads to podocyte apoptosis, GBM function change, filtration barrier damage, eventually lead to renal fibrosis. Some studies have confirmed that TGF-*β* is an important mediator causing progressive renal fibrosis and further developing into end-stage renal disease [41]. It is one of the important reasons for inducing CKD. In this study, the decrease of anti-PLA2R level in serum could indirectly explain the decrease of immune complex and the alleviation of renal tissue fibrosis, but whether it targets TGF-*β*1/Smad pathway needs further research.

This study investigated the effects of combination therapy with DHA and BMSCs on podocytes and renal fibrosis in mice with MN *in vivo* and *in vitro*. It was proved that combination therapy with DHA and BMSCs can improve podocyte injury and renal fibrosis in MN mice by downregulating TGF-*β*1/Smad pathway, so as to provide reference for the drug treatment of clinical MN. However, the specific mechanism of anti-PLA2R and fibrosis is still unclear and remains to be further explored.

## 5. Conclusion

The results showed that combination therapy with DHA and BMSCs ameliorated renal fibrosis in MN mice by protecting podocytes and inhibiting the TGF-*β*1/Smad signaling pathway.
